# The importance of education to increase the use of bed nets in villages outside of Kinshasa, Democratic Republic of the Congo

**DOI:** 10.1186/1475-2875-9-279

**Published:** 2010-10-12

**Authors:** Julie K Ndjinga, Noboru Minakawa

**Affiliations:** 1 Kinoise Clinic, AV.de La Justice N°36, Kinshasa, Democratic Republic of the Congo; 2 Department of Vector Ecology and Environment, Institute of Tropical Medicine (NEKKEN) and the Global COE Program, Nagasaki University, 1-12-4 Sakamoto, Nagasaki, Nagasaki 852-8523, Japan

## Abstract

**Background:**

Malaria is the most prominent disease in the Democratic Republic of the Congo (DRC), and long-lasting insecticide-treated nets (LLINs) have been distributed free of charge since 2006 to combat the disease. However, the success of this bed net campaign depends on sufficient bed net use in all age groups. This study was designed to examine the factors affecting bed net use in villages outside of Kinshasa.

**Methods:**

Two villages along the Congo River, totalling 142 households with 640 residents, were surveyed using a standard questionnaire. The interview determined the number, ages, and sexes of family members; the education level of the family head; the number, colour, and type of nets owned; and the number of nets used in the previous night. The size of house was also measured, and numbers of rooms and beds were recorded. These variables were examined to reveal important factors that affect bed net use.

**Results:**

A total of 469 nets were counted, and nearly all nets were white LLINs. Of these nets, 229 (48.8%) nets were used by 284 (44.4%) residents. Bed nets were used by over 90% of children 5 to 15 years of age, whereas less than 50% of the residents in other age groups used bed nets. The important variables affecting bed net use were numbers of beds and rooms in the house and the education level of the family head of household.

**Conclusion:**

Education was the most important factor affecting bed net use in the villages outside Kinshasa. Development of an educational programme, particularly one directed toward parents, is necessary to reduce misconceptions and increase prevalence of bed net use among all age groups.

## Background

Malaria is one of the leading causes of morbidity and mortality in the Democratic Republic of the Congo (DRC), with approximately 180,000 deaths attributed to malaria each year [1]. This is one fifth of the 863,000 malaria deaths reported worldwide by the World Health Organization in 2008 [2]. The large number of malaria cases in the DRC is due to high malaria transmission rates, and it is exacerbated by two decades of civil war that have decimated the health care infrastructure and the government's ability to deliver social services.

Insecticide-treated bed nets offer essential protection against mosquitoes and significantly reduce morbidity and mortality due to malaria, particularly in endemic areas [3,4]. The DRC government distributed nearly 11.2 million long-lasting insecticide-treated nets (LLINs) free of charge between 2006 and 2008. This campaign provided LLINs to only 30% of the households and 24% of children younger than 5 years old [5]. To boost bed net coverage, the National Malaria Control Programme (NMCP) in the DRC planned to distribute an additional 2 million LLINs to cover at least 4 million people in Kinshasa and its surrounding villages in 2008.

Attempts to use bed nets are often hampered by environmental, social, and cultural considerations. Potential factors that affect bed net use are education level of family head, wealth, colour and shape of bed nets, house structure, sleeping arrangement, distance to retail stores and cultural taboos [6-9]. Even though LLINs have gained popularity, the effectiveness of LLINs against malaria transmission will be impaired without their proper use. Studies have shown that owning LLINs does not always increase the prevalence of use [10-13]. For instance, nets are occasionally misused for other purposes such as fishing in villages near Lake Victoria [14]. Little information is available about bed net use in the DRC, with the exception of a few studies on bed net use by women and children in Kinshasa before the mass distribution [15-17]. This study was designed to investigate availability and use of bed nets after the recent mass distribution in the villages outside Kinshasa and to determine the factors affecting bed net use.

## Methods

### Study area

A field survey was conducted in two villages, Mombele and Mbangu Mbangu, along the Congo River, outside of Kinshasa in October and November 2009 at the beginning of the rainy season. Mombele and Mbangu Mbangu are approximately 60 km and 90 km away from Kinshasa. Malaria is endemic in these villages, with stable transmission throughout the year. *Plasmodium falciparum *is responsible for the serious forms of malaria, with *Anopheles gambiae *as the main vector of transmission [18]. The major income sources in the villages are fishing and traditional small-scale farming. Bed nets have been distributed by a local health centre within each village through the recent program organized by NMCP.

### Data collection

In total, 142 households with 640 residents were randomly selected, including 72 households in Mombele and 70 in Mbangu Mbangu. Oral informed consent to conduct interviews was obtained by the village chiefs and staff members of the Nsele District Department of Health. A responsible member of each household was interviewed in the local Lingala language using a standard questionnaire. Interviewees were asked the number, ages, and sexes of family members; the education level of the head of household; the number, colour, and type (LLIN or not) of nets they possessed; and the number of nets used in the previous night. They were also asked where the bed nets were obtained, and whether each family member had slept under a net either on a bed or on the floor the previous night. When any family members did not sleep under a net, interviewees were asked the most important reason for this choice. After the interview, the floor area (m^2^) of each house was measured using a tape measure, and the numbers of rooms and beds were counted. The locations of beds and nets were also noted.

### Data analysis

A generalized linear mixed model (GLMM) using the lme4 package in R with binomial distribution was used to examine whether bed net use was explained by residents age, sex, and village [19]. Two-way interaction terms were also included in the initial model. Age and sex were included in the initial model, because distributions of ITNs have been targeted to infants under 5 years of age and pregnant women [2]. As bed net use might differ between the villages, village was also included as a variable. Bed net use of each family member was a binary response variable defined as slept with or without a net. The backward selection procedure was modelled based on the Akaike Information Criteria and the log likelihood ratio test. Sampling dates were treated as a random intercept to consider variation between dates because the houses were surveyed on 16 separate dates. This modelling allowed for a random slope to find optimal random errors, if necessary.

The same modelling procedure was used to examine the relationships of bed net use at the household level with bed availability, bed net availability, house size, number of rooms, education level of head of household, and village as variables. Two-way interaction terms were also included in the initial model. Bed net use was a response variable defined as the ratio of the number of residents who slept with nets to the number of those who slept without nets in a house. The variables of bed availability, net availability, number of rooms and education level were included in the initial model, because past studies found that these variables affect bed net use [6-9]. As house size may affect bed availabily and number of rooms, this variable was also included. The variable of bed availability was defined as the total bed area (m^2^) in a house, and house size was reported as total floor area (m^2^). The values for these variables were divided by the adjusted number of residents in each house estimated based on body size: a child below 5 years of age was treated as 0.3 person, a child between 5 and 15 years of age as 0.5 person, and a person over 15 years of age as one person. The number of rooms in a house was not corrected for the number of residents because the qualitative characteristics of the rooms captured by the data might have been lost. For example, in a house with two or three rooms, one room was used as a living room and the others were usually used as bedrooms [9]. Using the raw number of rooms maintained both this characteristic and the quantitative characteristics. The education level was categorized by (1) incomplete primary education, (2) completed primary education, and (3) completed secondary or higher education.

## Results

In total, 640 residents and 469 nets were counted with an average of 4.7 residents (SD = 1.8) and 3.3 nets (SD = 1.3) per house. Of the 469 nets, 229 (48.8%) nets were used, and 284 (44.4%) residents had slept under nets in the previous night (Table 1). Thus, 0.7 nets were available per resident, and 1.2 residents slept under a net. The mean size of houses was 17.2 m^2 ^(SD = 5.0), and the mean number of rooms was 1.6 (SD = 0.7) and of beds was 1.6 (SD = 0.7). Of 142 head of households, 51 (35.9%) had not completed primary education, 43 (30.3%) had completed primary education, and 48 (33.8%) had completed secondary or higher education. Nearly all nets (468) were LLINs that were provided by the local health department free of charge and all nets were white.

**Table 1:** Numbers of residents who slept with nets and without nets.

	Slept with nets	Slept without nets	N
Sex			
Female	146 (42.2%)	200 (57.8%)	346
Male	138 (46.9%)	156 (53.1%)	294
Village			
Mombele	135 (40.4%)	199 (59.6%)	334
Mbangu Mbangu	149 (48.7%)	157 (51.3%)	306
Total	284 (44.4%)	356 (55.6%)	640

**Figure 1:**
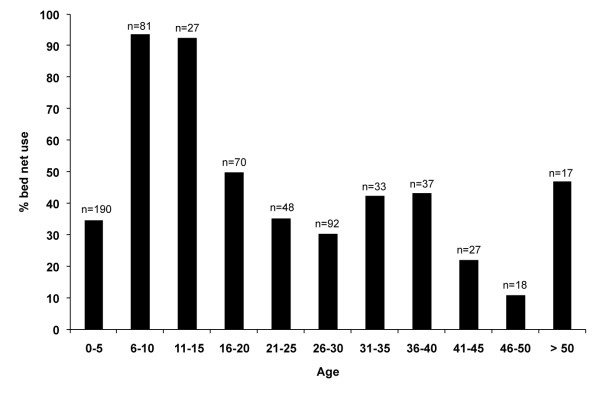
**Percentages of bed net use among different age groups**.

The GLMM revealed that age was significantly associated with individual bed net use, but the variables of sex and village were not associated (z = -5.09, p < 0.001). Bed nets were used by over 90% of children 5 to 15 years of age, whereas less than 50% of the residents in other age groups used bed nets (Figure 1).

**Figure 2:**
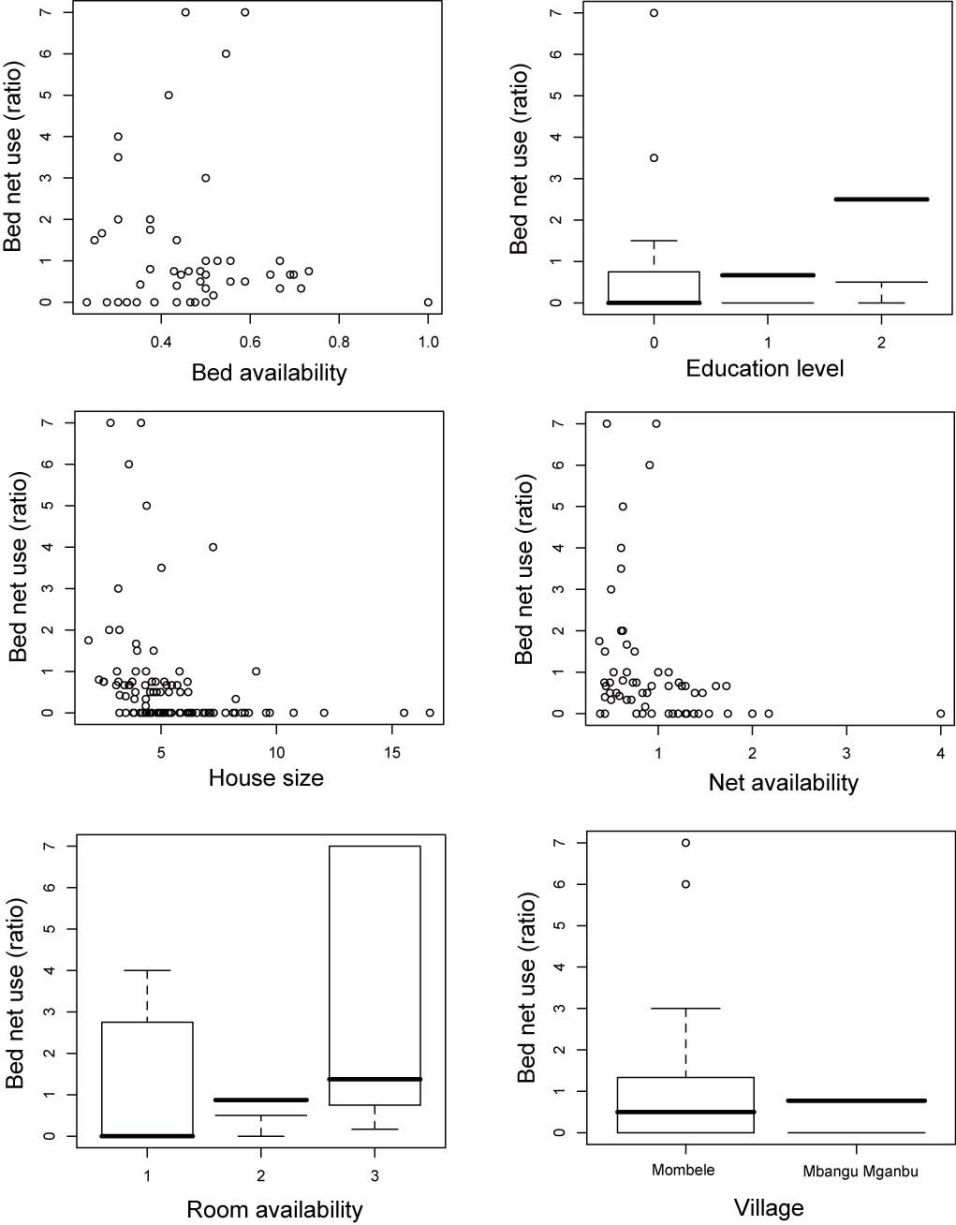
**Relationships of bed net use with bed availability, education level of family head, house size, net availability, room availability and village**. Bed net use was defined as the ratio of the number of residents who slept with nets to the number of those who slept without nets in a house

Household bed net use was examined using six variables: bed availability, bed net availability, house size, number of rooms, head of household's education level, and village (Figure 2). The three variables of net availability, house size, and village were not included in the final model. Bed availability (*z *= 3.77, *p *< 0.001), education level (*z *= 2.29, *p *= 0.022), and number of rooms (*z *= 3.81, *p *< 0.001) were significantly associated with bed net use at the household level. Net use was also significantly associated with the interaction between bed availability and the number of rooms (*z *= -2.56, *p *= 0.011).

A total of 66 interviewees provided reasons why they did not use bed nets. The most common reason (26 cases, 39.4%) was related to heat discomfort within a bed net. Twenty-two (33.3%) respondents believed that females become infertile under a bed net. Two other reasons included associating bed net use with sleeping in a coffin (nine cases, 13.7%) and the idea that the bed net campaign was a plot by the United States of America Central Intelligence Agency to reduce the African population by treating bed nets with a chemical that kills sleepers (eight cases, 12.1%).

## Discussion

The number of bed nets reported was enough to cover all the villagers, apparently a result of the recent NMCP campaign. Nevertheless, fewer than half of the nets were used, and half of the villagers did not sleep under the nets. A recent study reported that the prevalence of bed net use is 56% in Nigeria and over 90% in Senegal [10]. Thus, the proportion of nets used in the villages of the DRC is considerably lower compared with other African countries, and this may hamper the effectiveness of the malaria prevention campaign.

The most common reason given for not using bed nets was discomfort due to heat within the net. This reason was also common in other African countries [6,10,20,21]. The other reasons given were based on cultural myths, and failure to use bed nets is often associated with misconceptions and cultural taboos [22]. Discomfort due to the heat may also be a misconception; although the bed net may reduce airflow and increase temperature, the increase might not be noticeable, and if it is noticeable, one's health should not be comprised due to discomfort.

Education about malaria transmission and the benefits and proper use of bed nets should eliminate the misconceptions about bed nets usage. Results reported here demonstrate that the education level of the family head of household was associated with bed net use among family members in the surveyed villages. A previous study in Kinshasa also reported that women who had secondary school or higher education were 3.4 times more likely to own and 2.8 times more likely to use a bed net compared with women with less education [15]. A mother's education level and adequate knowledge about malaria transmission are also associated with their use of bed nets in other countries [21-23]. As a majority of children less than 5 years of age sleep with their parents in Africa, their protection from malaria depends on parents' perception of bed nets [24,25].

This study also found a lower prevalence of net use among children less than 5 years of age and among adults, and a greater prevalence of use among school children 5 to 15 years old. This result was unexpected, as several studies have reported lower use of bed net use among school children [9,21,25,26]. Mothers and infants are primary targets for net distribution, and their use of nets should be high [15,17]. In recent years, primary and secondary schools have focused on education in disease prevention and sanitation, including bed net use in the district where the surveyed villages in this study are located. This systematic education program may explain the high prevalence of nets use among children 5 and 15 years of age in these villages.

The other important factors associated with bed net use were the numbers of beds and rooms in the house. These results are comparable to those from other studies [21,27]. As the number of rooms increased, the role of each room became clearer: it is common to use one room as a living room when a house has more than two rooms, and the others as bedrooms [9,27]. Residents who sleep on the floor in living rooms would have less attachment to nets compared with nets hung over beds in bedrooms, as the living room nets are most likely taken down every morning. Consequently, having more bedrooms increases both privacy and the space available for beds, which in turn increases the number of sites that are suitable for hanging nets, thereby increasing net use.

## Conclusion

Assessed the reasons for not using bed nets, the greater use of nets among school children and the importance of education level of family head, it is evident that education was the most important factor affecting bed net use in the villages outside Kinshasa. Development of an educational programme, particularly one directed toward parents, is necessary to reduce misconceptions and increase prevalence of bed net use among all age groups.

## Competing interests

The authors declare that they have no competing interests.

## Authors' contributions

JNK conducted the fieldwork and drafted the first manuscript. NM conceived the study and finalized the manuscript. Both authors have read and approved the final manuscript.
